# Associations of Lifestyle and Dietary Factors with Urinary Bisphenol A, S, and F: Evidence from the Korean National Environmental Health Survey IV (2018–2020)

**DOI:** 10.3390/toxics13121027

**Published:** 2025-11-27

**Authors:** Se Ryeon Lee, Eun-Yeob Kim, Jaeyoung Kim

**Affiliations:** 1 Division of Oncology and Hematology, Department of Internal Medicine, Korea University Ansan Hospital, Ansan-si 15355, Republic of Korea; logost@korea.ac.kr; 2 Research Institute for Skin Image, Korea University College of Medicine, Seoul 08308, Republic of Korea; key0227@korea.ac.kr; 3 Department of Biomedical Sciences, College of Medicine, Korea University, Seoul 02841, Republic of Korea; 4 Core Research & Development Center, Korea University Ansan Hospital, Ansan-si 15355, Republic of Korea

**Keywords:** bisphenol, endocrine disruptors, lifestyle factors, dietary exposure, food packaging, environmental health, microplastics

## Abstract

Bisphenol A, Bisphenol S, and Bisphenol F are widely used plastic additives and endocrine-disrupting chemicals with potential adverse health effects. Limited research has examined lifestyle and dietary factors influencing human exposure to these compounds. This study investigated associations between urinary bisphenol concentrations and demographic, lifestyle, dietary, and food-handling factors in 4239 Korean adults aged 19 to 82 years (1889 men, 44.6%; 2350 women, 55.4%) from the Korean National Environmental Health Survey (2018–2020). Median urinary concentrations with interquartile ranges were 1.15 µg/L (IQR: 0.45–2.27) for BPA, 0.17 µg/L (IQR: 0.06–0.41) for BPF, and 0.15 µg/L (IQR: 0.06–0.38) for BPS. Urinary bisphenol concentrations differed significantly by gender, age, smoking status, alcohol consumption, and education level (*p* < 0.001), with higher concentrations in younger males, smokers, and alcohol consumers. Frequent consumption of instant noodles, microwaveable foods, canned foods, and plastic-wrapped takeout was significantly associated with elevated bisphenol levels (*p* < 0.001). Use of plastic containers, coated cookware, and electric rice cookers was linked to higher urinary BPA and BPS concentrations. BPA showed statistically significant but weak positive correlations with serum creatinine (r = 0.044, *p* < 0.05) and height (r = 0.037, *p* < 0.05), as assessed using Pearson’s correlation test. BPF was negatively correlated with aspartate aminotransferase (AST) levels (r = −0.042). Public health strategies should prioritize safer food storage practices and enhance awareness of health risks associated with these chemicals.

## 1. Introduction

Since the mass production of plastics began in the 1950s, they have been extensively utilized across various industries, including food packaging, construction materials, and machine components, due to their convenience and efficiency [1]. Globally, plastic production is projected to increase nearly threefold, rising from 322 million tons in 2015 to approximately 1 billion tons by 2050 [2,3]. Recently, concerns have grown over the exponential increase in plastic waste, which poses significant environmental threats through pollution and ecosystem disruption [3,4]. Microplastics, in particular, have garnered attention as a potential risk factor for food safety [4]. In South Korea, plastic waste generation increased from approximately 7.98 million tons in 2017 to 11.93 million tons in 2021, reflecting a sharp rise in household and business waste (Ministry of Environment Report) [3].

While plastic waste generation from construction debris decreased, it increased in household waste and waste from business facilities [5]. Currently, plastic waste is managed through incineration, landfilling, and recycling. As of 2017, 33.4% of total plastic waste was incinerated, 4.6% was landfilled, 39.3% was thermally recycled (energy recovery), and 22.7% was physically recycled [5,6]. However, incineration releases significant amounts of carbon dioxide and carcinogenic dioxins, while landfilling faces challenges including a shortage of landfill sites and soil contamination [7,8,9,10]. A domestic study estimated that potential microplastic generation from various waste processing facilities—including personal products, production and transportation processes, paint usage, household dust, outdoor dust, artificial turf, and food waste—ranges from 63,000 to 216,000 tons annually. This is 25-fold and 10-fold higher than in Norway and Sweden, respectively [11], but similar to the levels reported in Japan [12], which has a comparable population density and industrial structure to South Korea.

As global concerns about environmental pollution and human exposure to microplastics continue to escalate, the World Health Organization emphasized in 2019 the urgent need for research on the health risks associated with microplastics in drinking water [13]. Contamination of drinking water poses significant concerns, and the bioaccumulation of microplastics in seafood—including fish and shellfish—presents both physical and chemical risks to humans as the ultimate consumers in the food chain [13,14]. However, research on this topic remains insufficient. Among numerous plastic additives, this study focuses on bisphenols, a class of synthetic chemicals widely used in the production of polycarbonate plastics and epoxy resins for food and beverage containers, coated cookware, and can linings [15,16]. A well-known example of this group is Bisphenol A (BPA), one of the most widely produced and well-documented compounds with respect to human exposure and health concerns. Global BPA production exceeds several million tons annually, with Korea being a major producer and consumer in the plastics industry [15].

Extensive experimental and epidemiological evidence has identified BPA as an endocrine-disrupting chemical with estrogenic activity and potential links to adverse health outcomes [17,18]. As regulatory scrutiny has increased, manufacturers have increasingly adopted structural analogs—bisphenol S (BPS) and bisphenol F (BPF)—as BPA substitutes [16]. However, systematic evaluations indicate that BPS and BPF exhibit hormonal activities comparable to BPA, raising concerns that “regrettable substitution” may perpetuate similar biological risks [15,16]. Beyond toxicology, population biomonitoring demonstrates widespread exposure to BPA as well as its two analogs. BPS is consistently detected in urine samples across the United States and multiple Asian countries, and U.S. NHANES data confirm measurable urinary levels of BPA, BPS, and BPF in both adults and children [19]. Based on new toxicological data indicating potential adverse effects on the immune and endocrine systems, as well as evidence of widespread human exposure, the European Food Safety Authority (EFSA) substantially lowered the tolerable daily intake (TDI) for bisphenol A (BPA) in 2023 by a factor of approximately 20,000—from 4 µg/kg bw/day to 0.2 ng/kg bw/day. This revision highlights the public-health significance of evaluating the determinants of exposure to BPA and its analogs [20].

BPA, a plastic additive commonly found in microplastic-contaminated environments, can leach from plastic materials when heated and be absorbed by the human body, acting as an endocrine disruptor by mimicking estrogen and potentially interfering with hormone receptors [21,22]. Direct heating of canned food can release substantial amounts of BPA; therefore, transferring contents to another container after opening is advisable [21,22,23]. Additionally, thermal paper receipts contain BPA as a color developer, and in the presence of certain substances such as triclosan or triclocarban, BPA can leach out, making prolonged handling of receipts undesirable [20,21]. BPA is primarily excreted in urine after metabolism; a portion may remain or accumulate in tissues such as the liver and adipose tissue, depending on exposure duration and dose [24]. The chemical structures of BPA, BPF, and BPS all share the bisphenol backbone but differ in their bridging groups: isopropylidene in BPA, methylene in BPF, and sulfone in BPS [20]. These differences may influence their stability, environmental persistence, and endocrine-disrupting activities [23].

Therefore, this study aimed to investigate associations between demographic and lifestyle factors and exposure to BPA, as well as its structural analogs BPS and BPF. Additionally, we sought to identify which factors have the most significant impact on human exposure to these chemicals.

## 2. Materials and Methods

### 2.1. Study Design and Population

This study utilized data from the Korean National Environmental Health Survey Cycle 4 (KoNEHS IV, 2018–2020), conducted by the National Institute of Environmental Research under project number NIER-2018-01-01-001. The Cycle IV survey population was divided into three age groups—children/adolescents, adults, and the elderly—to assess environmental exposures across different life stages. The present analysis focused on adult participants aged ≥ 19 years who had available urinary bisphenol data (*n* = 4239; 1889 men and 2350 women). Individuals under 19 years of age were surveyed separately by the National Institute of Environmental Research and were not included in this study. Throughout this manuscript, the abbreviation KoNEHS is used to refer to the Korean National Environmental Health Survey. Participants were community-dwelling adults from the general population; no patients or specific occupational/high-exposure groups were included, such as workers engaged in plastic production and recycling plants, chemical synthesis laboratories, printing or coating industries, or medical facilities frequently using polycarbonate or epoxy-based equipment. Urine samples were collected as first-morning spot urine (approximately 20–50 mL) using sterile 125 mL capped polypropylene containers without preservatives at designated public health centers. Samples were immediately refrigerated at 4 °C, transported under cold-chain conditions within 24 h, and stored at −20 °C until analysis. The KoNEHS Cycle 4 received ethical approval from the International Review Board (IRB) of the National Institute of Environmental Research (NIER), Korea (IRB No. NIER-2018-BR-003-02). K-NEHES IV employed a nationally representative stratified, multistage probability sampling design to ensure representativeness of the Korean adult population. All analyses applied survey weights, stratification, and clustering variables provided in the dataset. Biomarker measurements, including BPA, BPF, and BPS, were conducted at the NIER Central Laboratory using liquid chromatography–tandem mass spectrometry (LC-MS/MS). Urinary bisphenols were extracted using a solid-phase extraction procedure with preconditioned cartridges, eluted with organic solvent, and concentrated under nitrogen prior to instrumental analysis. Quantification was performed using LC–MS/MS. The calibration range was 0.05–50 µg/L, and the calibration curves showed excellent linearity (R^2^ ≥ 0.999). The method detection limits (MDLs) were 0.05 μg/L for BPA, 0.05 μg/L for BPF, and 0.10 μg/L for BPS (Table 1). Method recoveries ranged from 95% to 105%, and accuracy and precision met NIER QA/QC criteria. Concentrations below the limit of quantification (LOQ) were substituted with LOQ divided by the square root of two (LOQ divided by √2) prior to statistical analysis. Details of method performance and non-detect handling are presented in Table 1. As KoNEHS IV is an open national dataset, the original measurements could not be modified by researchers. Instead, variable recoding, application of sample weights, and creation of derived variables (e.g., quartiles, dichotomization) were conducted according to the official KoNEHS IV Data User Guide published by the National Institute of Environmental Research in 2021. Only crude urinary bisphenol concentrations (μg/L) were used in the present analysis; creatinine-adjusted values were not applied.

### 2.2. Variables and Measurements

Study variables were categorized into four groups: (1) Demographic variables: gender, age, height (cm), weight (kg), waist circumference (cm), blood pressure (mmHg), education level, marital status, pregnancy status, and menopause status; (2) Lifestyle factors: alcohol consumption, smoking habits, exercise frequency, exercise duration, and exercise intensity; (3) Biochemical markers: urinary bisphenol concentrations (BPA, BPF, BPS), serum lipids (HDL cholesterol, total cholesterol, triglycerides), hepatic biomarkers (ALT, AST, GGT), and serum creatinine; (4) Dietary and food-handling practices: frequency of consumption of instant noodles, microwaveable packaged foods, canned foods, bottled beverages, plastic-wrapped delivery foods, disposable paper cups, and PET bottle beverages, as well as use of various food storage containers and cooking utensils. Only crude urinary concentrations (µg/L) were analyzed. Creatinine-adjusted values were not included in the final analysis because they were not required for the primary study objectives. In addition to exposure biomarkers, serum AST, ALT, and creatinine were treated as outcome variables in the weighted regression analyses to assess potential hepatic and renal effects of bisphenol exposure.

### 2.3. Statistical Analysis

Categorical variables were presented as frequencies and percentages, while continuous variables were expressed as means ± standard deviations. Normality of continuous variables was assessed using the Shapiro–Wilk test. When variables had more than two categories (e.g., education, age groups), overall differences were assessed using the Kruskal–Wallis test or Chi-square test for multiple groups. Correlation analyses between bisphenol concentrations and biochemical markers were performed using Spearman’s rank correlation coefficient. To identify the determinants of urinary BPA, BPF, and BPS concentrations, multivariable linear regression analyses were conducted, incorporating demographic, lifestyle, dietary, and food-handling variables. Subsequently, weighted linear regression models were developed to examine the associations between log-transformed bisphenol concentrations and hepatic biomarkers (AST, ALT) as well as serum creatinine. Weights were applied based on the complex sampling design of KoNEHS to ensure population-representative estimates. All models were adjusted for relevant covariates, including age, sex, BMI, smoking status, alcohol consumption, and education level. Regression coefficients (β) and *p*-values were reported for each bisphenol compound. All statistical analyses were conducted using IBM SPSS Statistics version 25.0 (IBM Corp., Armonk, NY, USA). Statistical significance was set at *p* < 0.05.

## 3. Results

### 3.1. General Characteristics of Study Participants

The study sample comprised 1889 males (44.6%) and 2350 females (55.4%). Mean daily exercise duration was 78.86 ± 48.05 min. Physical measurements revealed a mean height of 162.64 ± 8.91 cm, a weight of 65.90 ± 12.62 kg, and a waist circumference of 85.24 ± 10.41 cm. Mean systolic blood pressure was 132.70 ± 17.62 mmHg and diastolic blood pressure was 79.55 ± 12.43 mmHg. Median urinary bisphenol concentrations were: BPA 1.15 [0.45–2.27] µg/L, BPF 0.17 [0.06–0.41] µg/L, and BPS 0.15 [0.06–0.38] µg/L. According to the analytical guidelines of the KoNEHS, the reporting limits were 0.029 µg/L for BPA, 0.036 µg/L for BPF, and 0.022 µg/L for BPS. Based on these thresholds, detection frequencies were 95.0% for BPA, 85.7% for BPF, and 88.6% for BPS, respectively. These findings indicate that BPA was detected in nearly all urine samples, while BPF and BPS were also frequently detected, reflecting widespread exposure to bisphenol analogs among Korean adults. The concentration ranges were 0.05–80.3 µg/L for BPA, 0.05–20.4 µg/L for BPF, and 0.10–18.6 µg/L for BPS (Table 2). Serum lipid profiles showed a mean total cholesterol of 188.21 ± 38.04 mg/dL, HDL cholesterol of 54.75 ± 14.23 mg/dL, and triglycerides of 182.58 ± 149.03 mg/dL. Liver function enzymes were as follows: ALT 25.34 ± 16.44 U/L, AST 26.12 ± 9.62 U/L, and GGT 31.32 ± 39.78 U/L. Mean serum creatinine, an indicator of kidney function, was 0.79 ± 0.20 mg/dL. Detailed participant characteristics are presented in Supplementary Table S1.

### 3.2. Bisphenol Concentrations by Demographic Characteristics and Lifestyle Factors

Statistically significant differences were observed in all three bisphenol concentrations by gender (*p* < 0.001), with males exhibiting higher concentrations than females. Significant differences in BPA and BPS concentrations were also noted by age (*p* < 0.001), with higher levels in younger age groups. Current smokers showed significantly higher urinary concentrations of BPA (*p* < 0.001), BPF (*p* < 0.001), and BPS (*p* = 0.008) compared to non-smokers. Alcohol consumption was associated with significantly elevated BPA and BPS concentrations (*p* < 0.001), with alcohol consumers exhibiting higher levels than non-consumers. Education level demonstrated significant associations with BPA and BPS concentrations (*p* < 0.001), with concentrations varying across educational categories (Table 2).

### 3.3. Associations Between Dietary Factors and Urinary Bisphenol Concentrations

Significant associations were identified between bisphenol concentrations and various dietary factors, including frequency of instant noodle consumption, microwaveable packaged food intake, canned food consumption, plastic-wrapped delivery food, disposable paper cup use, and bottled beverage consumption (*p* < 0.001 or *p* < 0.05). Higher frequencies of instant noodle (≥3 times/week), microwaveable packaged food, and canned food consumption were particularly associated with elevated BPA concentrations (*p* < 0.001), and BPS concentrations also showed significant increases among frequent consumers (*p* = 0.02–0.04). Frequent use of vinyl-wrapped delivery foods and disposable paper cups was associated with increased BPA levels (*p* < 0.05), while bottled beverage consumption was linked to higher BPA and BPF concentrations (*p* < 0.05). Detailed results of these associations are shown (Table 3).

### 3.4. Associations Between Food Storage Practices, Cooking Utensils, and Urinary Bisphenol Concentrations

Urinary bisphenol concentrations varied significantly according to food storage practices and cooking utensil usage patterns. Participants who reported frequent use of plastic containers had significantly higher BPA concentrations (*p* < 0.001), while frequent use of coated cookware and electric rice cookers was associated with elevated BPA and BPS concentrations (*p* < 0.05). No significant differences were observed for BPF according to these practices (Table 4). BPS concentrations differed significantly by freezer storage container type (*p* < 0.001), with higher concentrations observed when using plastic bags or zip bags. The frequency of plastic container use for storing cooked food was significantly associated with BPF (*p* = 0.009) and BPS (*p* < 0.001) concentrations, with increasing concentrations observed with higher usage frequency. Storing boiled water in plastic containers was associated with elevated BPF (*p* = 0.006) and BPS (*p* < 0.001) concentrations. Use of coated cookware showed significant associations with bisphenol concentrations: coated frying pans with BPA (*p* = 0.008) and BPS (*p* = 0.001), and coated pots with both BPA and BPS (*p* < 0.001). Frequency of coated rice cooker and pressure cooker use was associated with BPA (*p* = 0.014) and BPS (*p* < 0.001) concentrations. BPF concentrations showed limited associations, primarily with specific plastic container usage (*p* = 0.009 and *p* = 0.006) (Table 4 and Table 5).

### 3.5. Correlations Between Urinary Bisphenol Concentrations and Biochemical and Anthropome-Tric Indicators

Correlation analyses between urinary bisphenol concentrations and biochemical and anthropometric indicators revealed several significant associations. BPA showed significant positive correlations with BPS (r = 0.037, *p* < 0.05), serum creatinine (r = 0.044, *p* < 0.05), height (r = 0.037, *p* < 0.05), and weight (r = 0.033, *p* < 0.05). Conversely, BPA demonstrated a significant negative correlation with systolic blood pressure (r = −0.048, *p* < 0.01). Urinary BPF concentrations were significantly lower in menopausal women compared to non-menopausal women (*p* = 0.001). Significant differences were also observed across marital status categories (*p* < 0.001), with the lowest median levels found among divorced or separated individuals (Table 2).

### 3.6. Associations Between Bisphenol Exposure and Hepatic Biomarkers

No significant correlations were observed between BPA and serum total cholesterol, triglycerides, or liver enzymes. BPF showed weak, non-significant correlations with BPA (r = 0.009) and BPS (r = 0.019). A significant negative correlation was found between BPF and AST (r = −0.042, *p* < 0.05), suggesting an inverse association between BPF levels and liver enzyme activity (Table 6). Weighted linear regression analyses (Table 7) revealed significant positive associations between log-transformed bisphenol concentrations and hepatic biomarkers. Specifically, log-transformed BPA levels were significantly associated with increased serum AST (β = 0.123, *p* = 0.003) and ALT (β = 0.176, *p* = 0.001). Similarly, BPS showed positive relationships with both AST (β = 0.097, *p* = 0.004) and ALT (β = 0.124, *p* = 0.007), suggesting potential hepatocellular effects. In contrast, BPF exhibited only marginal associations with these hepatic enzymes, indicating a comparatively weaker influence. When BPA, BPS, and BPF were simultaneously included in the multivariable model (Table 8), only BPA retained significant associations with both AST and ALT after adjusting for potential confounders, including demographic and lifestyle factors.

The results of the weighted regression analysis are presented in Table 7 and Table 8. BPA exposure was found to be significantly associated with elevated liver enzyme levels, whereas BPF and BPS showed no significant associations. These findings suggest that the hepatotoxic potential of BPA remains greater than that of its analogs. When considered alongside the data in Table 4 and Table 5, it is evident that frequent consumption of canned and instant foods is linked to higher BPA exposure, which in turn leads to increased AST and ALT levels.

## 4. Discussion

Concentrations of all three bisphenol compounds differed significantly by gender, age, smoking status, and alcohol consumption, which may reflect differences in dermal and occupational exposure patterns [25]. Liao and Kannan (2014) reported higher BPA levels in men than women and greater exposure tendencies in younger individuals [26], findings that our study confirmed. Current smokers and alcohol consumers demonstrated elevated BPA and BPS concentrations, potentially linked to increased use of disposable products and packaging materials containing bisphenols. Regarding dietary factors, frequent consumption of instant noodles, microwaveable foods, canned foods, plastic-wrapped delivery meals, and bottled beverages was significantly correlated with elevated BPA and BPS levels. These findings align with Rudel et al. (2011), who reported that consumption of plastic and canned packaged foods elevates BPA concentrations [27].

Our multivariable regression analyses identified canned food consumption as a key determinant of urinary BPA levels, consistent with evidence of bisphenol leaching from can linings [28,29]. Lower BPF levels among menopausal women may reflect hormonal or behavioral differences in exposure patterns [16]. Additionally, lower BPS levels among married participants could be linked to dietary or lifestyle factors not fully captured in our models. Importantly, although we examined multiple packaging and utensil-related variables (e.g., PET bottles, disposable paper cups, coated cookware), most were not significantly associated with bisphenol exposure in this nationally representative sample. This finding underscores the importance of dietary sources, particularly canned foods, as the dominant contributors to bisphenol exposure in the Korean population.

Use of coated cooking utensils and tableware was associated with bisphenol exposure. Frequent use of plastic containers, coated frying pans, pots, and electric rice cookers affected BPA and BPS concentrations, as these compounds may leach during high-temperature cooking. Our findings suggest that bisphenols can be absorbed by the body through plastic containers or coated products exposed to elevated temperatures. The choice and frequency of container use during food storage and cooking significantly influenced bisphenol exposure levels. Therefore, primary factors contributing to bisphenol exposure are closely linked to food packaging materials and household items, indicating that reducing the use of these products or adopting alternatives may help decrease exposure [30].

BPA showed significant positive correlations with serum creatinine, height, and weight, while demonstrating a negative correlation with systolic blood pressure. These associations suggest that BPA exposure may influence renal function or metabolic regulation. Additionally, BPF displayed a significant negative correlation with AST levels, indicating a potential relationship with liver enzyme activity. These findings demonstrate that bisphenol exposure is not merely an environmental concern but is closely linked to individual lifestyle, dietary habits, and cooking methods, with potential correlations to specific physiological indicators [31,32]. Notably, such exposure could have cumulative health effects over time, underscoring the need for comprehensive mechanistic studies. Based on these biochemical correlations, the results of further analyses on the effects of bisphenol exposure on the liver are as follows. The present findings extend previous research by demonstrating that BPA, rather than its structural analogs BPF and BPS, exhibits the strongest association with hepatic biomarkers after controlling for demographic factors (Table 5). These results align with toxicological evidence indicating that BPA can induce oxidative stress and hepatocellular injury through endocrine-disrupting pathways [31]. Although BPF and BPS have been introduced as “safer” substitutes, their weaker or nonsignificant associations with AST and ALT suggest that their hepatotoxic potential may be lower—or that current exposure levels remain insufficient to reveal measurable effects. Integrating these outcomes with lifestyle and dietary analyses (Table 5 and Table 6) reveals that individuals who frequently consume canned or instant foods and have higher rates of smoking and alcohol use exhibit elevated BPA levels, which, in turn, are associated with increased liver enzyme concentrations.

Our findings reaffirm that environmental bisphenol exposure sources are diverse and widespread [16]. In modern society, where convenience food consumption and disposable packaging use are increasing, seemingly simple food choices can result in endocrine-disrupting chemical exposure. This highlights the necessity for both consumer behavior modification and policy interventions at the manufacturing level. Furthermore, exposure to BPA alternatives such as BPF and BPS has become prevalent, with BPS showing particularly high concentrations associated with instant noodles, canned foods, and coated cookware. This situation represents “regrettable substitution,” where alternative substances introduced following BPA regulation may pose new health risks [33].

Correlation analyses with biochemical indicators are insufficient to establish causal relationships. However, observed associations with serum creatinine and systolic blood pressure highlight the need for further research into physiological pathways through which bisphenol compounds may influence metabolism, renal function, and blood pressure regulation. Long-term cohort studies are necessary to clarify relationships between chronic diseases such as hypertension and metabolic syndrome and bisphenol exposure. Additionally, socioeconomic status and education level influenced exposure concentrations, indicating that environmental justice perspectives should be incorporated into discussions. Vulnerable populations likely have higher processed food consumption and disposable product use, potentially leading to health inequalities; therefore, tailored environmental health management strategies based on socioeconomic factors are required [34]. Currently, there is a lack of long-term monitoring systems and standards for BPF and BPS, requiring urgent systematic responses. BPS has demonstrated higher in vivo stability and potential toxicity than BPA in several studies, necessitating proactive public health measures.

However, not all plastic products contain BPA, and endocrine disruptors can be found in various media, including air, water, and soil [35]. Furthermore, BPA leaches from plastic products only under specific conditions; therefore, generalized safety concerns may be excessive. In 2008, the U.S. National Toxicology Program acknowledged potential risks to fetuses, infants, and children but concluded that typical environmental exposure levels do not significantly impact human health. The U.S. Food and Drug Administration (FDA) stated that BPA detected in food occurs in extremely low amounts and is rapidly metabolized and excreted, posing minimal risk [36]. The European Food Safety Authority (EFSA) determined in 2015 that the likelihood of BPA causing human health issues is low [37]. South Korea recommended a significant reduction in BPA standard limits in 2024, from 4000 ng/kg body weight per day to 0.2 ng/kg body weight per day [38]. This represents a precautionary measure that effectively promotes BPA market withdrawal.

This study has several limitations that should be acknowledged. First, the cross-sectional design precludes establishing causal relationships between bisphenol exposure and health outcomes. The temporal sequence of exposure and biochemical changes cannot be determined from our data. Second, dietary and lifestyle information was collected through self-reported surveys, which may be subject to recall bias and social desirability bias, potentially affecting the accuracy of exposure assessments. Third, bisphenol concentrations were measured from single urine samples, which may not accurately reflect long-term or chronic exposure patterns due to the relatively short half-life of these compounds in the body. Fourth, limited existing research on BPF and BPS exposure, combined with less established analytical criteria compared to BPA, constrains the interpretation and comparison of our findings. Fifth, we did not control for exposure to other environmental contaminants such as phthalates, parabens, and other endocrine-disrupting chemicals, which may act as confounding factors or interact synergistically with bisphenols. Finally, the study population was limited to Korean adults, which may limit the generalizability of findings to other populations with different dietary patterns, lifestyle factors, and regulatory environments.

These findings are in line with previous studies; for example, Liao and Kannan (2014) reported higher BPA levels in men than women and greater exposure tendencies in younger individuals in China [26]. Similarly, Rudel et al. (2011) observed that consumption of plastic and canned packaged foods elevated BPA concentrations in the USA [27]. Although bisphenol exposure has been studied in both Korean children and adolescents, the KoNEHS for adults and the KoNEHS for children and adolescents are conducted as separate national survey systems with distinct sampling frames and exposure assessment methodologies. Therefore, direct comparisons between these two datasets are not appropriate. Despite these limitations, this study provides valuable insights into the associations between lifestyle factors and bisphenol exposure in a large, representative sample of Korean adults, contributing to the growing body of evidence on environmental health risks associated with plastic-derived chemicals.

## 5. Conclusions

This study comprehensively examined the associations between urinary concentrations of BPA, BPF, and BPS and a broad range of demographic, lifestyle, and dietary factors, as well as their relationships with biochemical and anthropometric indicators in a nationally representative sample of Korean adults. Bisphenol concentrations varied significantly by gender, age, smoking status, and alcohol consumption, with higher exposure observed among younger males, smokers, and alcohol consumers. Dietary habits emerged as the strongest determinant of bisphenol burden: frequent consumption of canned and instant foods, along with the use of plastic containers and coated cookware during food storage and preparation, was strongly associated with elevated urinary BPA and BPS concentrations. These findings underscore the critical role of everyday food-related behaviors in influencing internal bisphenol exposure.

Importantly, BPA demonstrated significant positive associations with hepatic biomarkers (AST, ALT) and serum creatinine, suggesting potential hepatic and renal effects. In contrast, BPF and BPS exhibited weaker or non-significant associations. This pattern supports the concept of “regrettable substitution,” indicating that BPA remains the predominant hepatotoxic agent among bisphenol analogs. Therefore, reducing BPA exposure through behavioral modifications—such as limiting consumption of canned and processed foods, avoiding microwaving plastic containers, and using safer cookware—may represent an effective public health strategy for mitigating hepatic dysfunction.

Overall, our findings emphasize the need for stricter regulatory controls on bisphenol use in food packaging, the development of safer chemical alternatives, and public education on safe food handling practices. Enhanced biomonitoring systems and targeted interventions for high-risk groups—particularly young men and frequent consumers of convenience foods—are also warranted. Future longitudinal studies should further elucidate the causal pathways linking bisphenol exposure to metabolic and hepatic disorders and assess the cumulative effects of mixed bisphenol exposures in real-world settings.

## Figures and Tables

**Table 1:** Method performance and non-detect handling for urinary bisphenols.

Chemical	MDL (μg/L)	Calibration R^2^	Check Standard Acceptance	Non-Detect Rule	Detection Rate (%)	LOQ (µg/L)
BPA	0.05	≥0.999	±10% per~20 samples	MDL/√2	95.0	0.029
BPF	0.05	≥0.999	±10% per~20 samples	MDL/√2	85.7	0.036
BPS	0.10	≥0.999	±10% per~20 samples	MDL/√2	88.6	0.022

**Table 2:** Urinary bisphenol concentrations by demographic and lifestyle characteristics.

Variable		Bisphenol A (Urine, µg/L)				Bisphenol F (Urine, µg/L)				Bisphenol S (Urine, µg/L)			
		M	SD	Median (25–75%)	*p*-Value	M	SD	Median (25–75%)	*p*-Value	M	SD	Median (25–75%)	*p*-Value
Gender	Male	2.70	6.47	1.30 [0.57–2.54]	<0.001	0.64	2.55	0.20 [0.06–0.48]	<0.001	0.59	2.24	0.16 [0.07–0.40]	<0.001
	Female	2.13	5.56	1.02 [0.37–2.04]		0.77	5.62	0.14 [0.06–0.35]		0.52	2.41	0.14 [0.05–0.35]	
Age	19–29 years	2.51	4.59	1.37 [0.64–2.65]	<0.001	1.01	4.89	0.16 [0.06–0.42]	0.589	0.72	1.95	0.19 [0.07–0.55]	<0.001
	30–39 years	2.87	6.89	1.31 [0.51–2.74]		1.17	6.72	0.18 [0.06–0.44]		0.75	3.08	0.16 [0.07–0.46]	
	40–49 years	2.38	4.79	1.24 [0.52–2.41]		0.90	7.27	0.15 [0.06–0.39]		0.50	1.52	0.16 [0.07–0.42]	
	50–59 years	2.69	8.63	1.13 [0.49–2.25]		0.59	2.53	0.16 [0.06–0.37]		0.55	2.06	0.15 [0.06–0.37]	
	60–69 years	2.12	4.76	0.95 [0.38–1.98]		0.47	1.59	0.18 [0.06–0.40]		0.43	1.42	0.14 [0.06–0.33]	
	70+ years	1.75	3.49	0.95 [0.39–1.82]		0.40	0.94	0.18 [0.06–0.43]		0.49	3.89	0.12 [0.05–0.26]	
Marital Status	Single	2.64	5.27	1.33 [0.53–2.59]	0.004	1.17	6.62	0.17 [0.06–0.42]	0.865	0.76	2.47	0.18 [0.07–0.53]	<0.001
	Married/Cohabiting	2.37	6.08	1.15 [0.45–2.18]		0.66	4.35	0.17 [0.06–0.40]		0.54	2.47	0.15 [0.06–0.37]	
	Divorced/Separated	2.20	6.21	0.98 [0.41–2.26]		0.48	1.42	0.16 [0.06–0.40]		0.34	0.67	0.13 [0.05–0.31]	
Pregnancy	No	2.23	4.37	1.12 [0.45–2.16]	0.113	1.37	8.56	0.14 [0.06–0.38]	0.967	0.84	2.95	0.16 [0.05–0.51]	0.100
	Yes	2.11	5.73	1.00 [0.36–2.02]		0.67	4.95	0.14 [0.06–0.35]		0.46	2.30	0.13 [0.06–0.33]	
Menopausal Status	No	2.23	4.78	1.17 [0.43–2.22]	<0.001	1.15	8.16	0.13 [0.06–0.34]	0.011	0.58	1.93	0.16 [0.06–0.44]	0.001
	Yes	2.05	6.09	0.91 [0.33–1.91]		0.47	2.05	0.16 [0.06–0.36]		0.47	2.73	0.12 [0.05–0.30]	
Smoking Status	Never smoked	2.30	6.12	1.08 [0.42–2.12]	<0.001	0.73	4.54	0.15 [0.06–0.37]	<0.001	0.53	2.37	0.14 [0.06–0.37]	0.008
	Former smoker	2.39	5.79	1.23 [0.55–2.42]		0.72	5.32	0.18 [0.06–0.40]		0.55	2.58	0.15 [0.06–0.37]	
	Current smoker	2.72	5.66	1.32 [0.52–2.53]		0.65	3.22	0.23 [0.06–0.55]		0.62	1.84	0.17 [0.07–0.42]	
Alcohol consumption	No	2.08	4.67	0.91 [0.31–2.10]	<0.001	0.48	1.56	0.15 [0.06–0.36]	0.275	0.40	1.23	0.12 [0.05–0.31]	<0.001
	Yes	2.46	6.27	1.20 [0.50–2.30]		0.77	4.99	0.17 [0.06–0.42]		0.59	2.53	0.16 [0.07–0.40]	
Final education level	No formal education	1.44	2.82	0.91 [0.38–1.41]	<0.001	0.30	0.31	0.19 [0.06–0.41]	0.850	0.22	0.50	0.09 [0.02–0.20]	<0.001
	Elementary school	1.88	4.46	0.94 [0.33–1.90]		0.63	2.61	0.18 [0.06–0.42]		0.52	3.99	0.11 [0.05–0.26]	
	Middle school	2.14	4.13	0.94 [0.37–1.98]		0.41	1.09	0.17 [0.06–0.40]		0.39	1.10	0.12 [0.05–0.28]	
	High school	2.45	6.56	1.15 [0.49–2.35]		0.57	2.87	0.17 [0.06–0.41]		0.57	1.86	0.16 [0.07–0.42]	
	University (less than 4 years)	2.78	5.90	1.34 [0.57–2.57]		0.47	1.23	0.16 [0.06–0.42]		0.70	2.51	0.19 [0.07–0.46]	
	University (4 years or more)	2.37	4.75	1.22 [0.52–2.37]		1.06	6.87	0.17 [0.06–0.39]		0.55	2.23	0.15 [0.07–0.41]	
	Graduate school	3.05	11.90	1.19 [0.50–2.35]		1.42	9.43	0.15 [0.06–0.39]		0.53	1.42	0.17 [0.07–0.41]	
Sweat-inducing exercise	No	2.48	6.81	1.12 [0.46–2.25]	0.696	0.73	4.32	0.16 [0.06–0.40]	0.420	0.56	2.71	0.14 [0.06–0.36]	0.012
	I exercise, but not enough to sweat	2.04	2.99	1.24 [0.49–2.31]		0.91	6.94	0.20 [0.06–0.45]		0.54	1.44	0.19 [0.08–0.43]	
	Yes	2.35	5.43	1.14 [0.44–2.27]		0.63	3.91	0.17 [0.06–0.40]		0.54	1.97	0.15 [0.06–0.39]	
Average weekly exercise frequency	1–2 times per week	2.14	3.44	1.17 [0.49–2.37]	0.905	0.57	1.46	0.19 [0.06–0.41]	0.265	0.52	1.40	0.16 [0.06–0.43]	0.659
	3–4 times per week	2.59	7.32	1.16 [0.45–2.30]		0.57	1.92	0.17 [0.06–0.40]		0.58	2.36	0.15 [0.06–0.39]	
	5–6 times per week	2.27	4.17	1.15 [0.39–2.34]		1.03	8.12	0.16 [0.06–0.42]		0.56	2.35	0.14 [0.06–0.35]	
	Almost every day	2.31	4.77	1.10 [0.44–2.06]		0.43	1.52	0.16 [0.06–0.38]		0.47	1.40	0.15 [0.06–0.35]	

**Table 3:** Urinary bisphenol concentrations by frequency of processed food consumption.

Variable		Bisphenol A (Urine, µg/L)				Bisphenol F (Urine, µg/L)				Bisphenol S (Urine, µg/L)			
		M	SD	Median (25–75%)	*p*-Value	M	SD	Median (25–75%)	*p*-Value	M	SD	Median (25–75%)	*p*-Value
Cup Ramen	Rarely	2.27	6.11	1.05 [0.42~2.06]	<0.001	0.67	4.02	0.16 [0.06~0.39]	0.423	0.48	2.37	0.13 [0.06~0.33]	<0.001
	Once a month	2.84	7.29	1.25 [0.49~2.59]		0.67	2.19	0.20 [0.06~0.45]		0.60	1.98	0.17 [0.07~0.43]	
	2–3 times a month	2.37	4.43	1.33 [0.49~2.62]		0.89	7.09	0.17 [0.06~0.41]		0.76	2.95	0.19 [0.08~0.51]	
	Once a week	2.52	5.19	1.28 [0.65~2.48]		0.58	1.54	0.16 [0.06~0.40]		0.58	1.87	0.15 [0.07~0.41]	
	2–3 times a week	2.27	3.47	1.38 [0.63~2.42]		1.16	8.53	0.18 [0.06~0.39]		0.71	2.13	0.21 [0.07~0.58]	
	4–6 times a week	3.84	11.09	1.36 [0.62~2.43]		0.59	1.45	0.12 [0.03~0.53]		0.41	0.56	0.19 [0.10~0.51]	
	Once a day	1.61	1.54	1.01 [0.56~2.03]		0.25	0.26	0.08 [0.06~0.41]		0.57	0.70	0.23 [0.12~0.84]	
	Twice a day	5.37		5.37 [5.37~5.37]		0.03		0.03 [0.03~0.03]		2.57		2.57 [2.57~2.57]	
Microwave-Packaged Food	Rarely	2.35	6.63	1.05 [0.42~2.11]	<0.001	0.68	4.66	0.16 [0.06~0.39]	0.318	0.47	2.33	0.13 [0.06~0.32]	<0.001
	Once a month	2.49	4.85	1.23 [0.54~2.42]		0.49	1.49	0.17 [0.06~0.38]		0.65	2.07	0.19 [0.08~0.43]	
	2–3 times a month	2.45	4.25	1.28 [0.54~2.41]		0.67	2.23	0.21 [0.06~0.48]		0.78	3.23	0.19 [0.08~0.46]	
	Once a week	2.31	3.39	1.38 [0.54~2.68]		1.49	8.24	0.19 [0.06~0.42]		0.59	1.46	0.18 [0.07~0.51]	
	2–3 times a week	2.23	3.76	1.22 [0.52~2.38]		0.58	1.93	0.15 [0.05~0.43]		0.78	2.08	0.18 [0.07~0.53]	
	4–6 times a week	2.67	3.61	1.36 [0.93~2.56]		0.61	1.48	0.13 [0.06~0.35]		1.00	1.99	0.31 [0.10~0.84]	
	Once a day	4.66	13.13	1.44 [0.63~2.85]		0.51	0.89	0.19 [0.08~0.35]		0.29	0.40	0.12 [0.07~0.4]	
	Twice a day	2.33	1.64	2.25 [1.20~3.27]		0.36	0.43	0.16 [0.03~0.66]		0.73	0.54	0.64 [0.26~1.25]	
	Three a day	0.33		0.33 [0.33~0.33]		0.48		0.48 [0.48~0.48]		0.02		0.02 [0.02~0.02]	
Canned Food	Rarely	2.22	6.17	0.97 [0.42~2.02]	<0.001	0.54	1.93	0.17 [0.06~0.40]	0.324	0.54	2.76	0.14 [0.05~0.35]	<0.001
	Once a month	2.12	4.49	1.15 [0.43~2.12]		0.87	5.82	0.18 [0.06~0.43]		0.51	1.97	0.14 [0.06~0.34]	
	2–3 times a month	1.96	2.95	1.22 [0.48~2.26]		0.77	4.18	0.17 [0.06~0.39]		0.63	2.55	0.16 [0.07~0.44]	
	Once a week	2.58	4.68	1.25 [0.50~2.58]		1.02	7.52	0.16 [0.06~0.41]		0.49	1.34	0.16 [0.07~0.37]	
	2–3 times a week	2.99	5.36	1.40 [0.68~3.13]		0.83	6.69	0.15 [0.06~0.41]		0.53	1.26	0.18 [0.07~0.43]	
	4–6 times a week	5.53	17.77	1.44 [0.75~3.04]		0.68	1.86	0.19 [0.06~0.49]		0.59	1.72	0.18 [0.07~0.50]	
	Once a day	5.35	15.44	1.93 [0.88~4.05]		0.46	1.24	0.13 [0.06~0.36]		0.60	0.86	0.21 [0.08~0.87]	
	Twice a day	5.15	6.54	2.18 [1.07~5.37]		0.16	0.17	0.12 [0.03~0.24]		1.43	2.61	0.31 [0.16~1.33]	
	Three a day	7.88	16.37	0.91 [0.27~1.05]		0.09	0.09	0.06 [0.03~0.10]		0.34	0.38	0.2 [0.13~0.27]	
Vinyl-Wrapped Food	Rarely	2.41	6.58	1.15 [0.45~2.26]	0.490	0.66	3.93	0.17 [0.06~0.39]	0.489	0.57	2.11	0.15 [0.06~0.39]	0.007
	Once a month	2.48	4.04	1.19 [0.54~2.48]		0.77	3.76	0.21 [0.06~0.41]		0.64	1.83	0.21 [0.07~0.43]	
	2–3 times a month	1.96	2.34	1.25 [0.57~2.28]		0.56	1.84	0.20 [0.06~0.39]		0.59	1.79	0.17 [0.07~0.50]	
	Once a week	1.95	3.17	1.32 [0.55~2.27]		1.59	10.30	0.13 [0.06~0.42]		0.47	1.19	0.16 [0.07~0.40]	
	2–3 times a week	2.75	6.16	1.15 [0.53~2.28]		0.67	2.95	0.17 [0.06~0.41]		0.40	0.90	0.14 [0.06~0.33]	
	4–6 times a week	2.65	6.96	1.19 [0.41~2.58]		0.40	0.79	0.19 [0.06~0.41]		0.36	0.57	0.17 [0.08~0.42]	
	Once a day	2.22	4.73	0.98 [0.40~2.10]		0.83	6.42	0.15 [0.06~0.46]		0.57	4.18	0.12 [0.05~0.30]	
	Twice a day	2.25	3.50	1.24 [0.49~2.35]		0.83	2.64	0.23 [0.09~0.51]		0.49	1.83	0.13 [0.05~0.26]	
	Three a day	2.89	4.29	1.42 [0.83~3.04]		0.17	0.16	0.14 [0.06~0.24]		0.21	0.19	0.12 [0.07~0.40]	

**Table 4:** Urinary bisphenol concentrations by frequency of food packaging material use.

Variable		Bisphenol A (Urine, µg/L)				Bisphenol F (Urine, µg/L)				Bisphenol S (Urine, µg/L)			
		M	SD	Median (25–75%)	*p*-Value	M	SD	Median (25–75%)	*p*-Value	M	SD	Median (25–75%)	*p*-Value
Delivery food packed with plastic wrap	Rarely	2.33	6.04	1.04 [0.42~2.09]	<0.001	0.50	2.29	0.16 [0.06~0.38]	0.628	0.50	2.55	0.13 [0.05~0.34]	<0.001
	Once a month	2.48	5.18	1.19 [0.51~2.31]		0.94	6.11	0.17 [0.06~0.44]		0.55	1.78	0.16 [0.07~0.39]	
	2–3 times a month	2.50	6.57	1.26 [0.44~2.46]		0.88	6.05	0.20 [0.06~0.46]		0.55	1.42	0.17 [0.07~0.44]	
	Once a week	2.73	7.79	1.40 [0.64~2.77]		1.48	9.08	0.18 [0.06~0.38]		0.60	1.67	0.16 [0.07~0.42]	
	2–3 times a week	1.96	2.20	1.43 [0.66~2.49]		0.91	3.86	0.15 [0.06~0.48]		1.01	4.07	0.26 [0.10~0.65]	
	4–6 times a week	1.85	2.57	0.98 [0.53~2.30]		0.45	0.96	0.18 [0.03~0.29]		0.54	0.63	0.28 [0.15~0.72]	
	Once a day	2.54	3.43	1.38 [0.61~3.07]		1.25	3.50	0.15 [0.04~0.52]		0.87	2.14	0.25 [0.10~0.59]	
	Twice a day	1.36	0.20	1.36 [1.22~1.51]		0.28	0.14	0.28 [0.18~0.37]		0.57	0.55	0.57 [0.18~0.95]	
	Three a day	1.67	0.82	1.60 [0.89~2.52]		0.30	0.07	0.34 [0.22~0.34]		2.57	2.29	3.06 [0.08~4.58]	
Use of disposable paper cups	Rarely	2.16	5.15	1.03 [0.38~2.10]	0.003	0.67	3.13	0.17 [0.06~0.40]	0.174	0.52	2.76	0.13 [0.05~0.36]	<0.001
	Once a month	1.85	3.12	0.94 [0.46~1.92]		0.84	5.85	0.18 [0.06~0.45]		0.74	2.81	0.16 [0.07~0.46]	
	2–3 times a month	2.16	4.16	1.07 [0.43~2.14]		0.65	1.96	0.20 [0.06~0.46]		0.80	3.69	0.18 [0.07~0.42]	
	Once a week	2.44	6.47	1.19 [0.53~2.23]		1.50	11.72	0.17 [0.06~0.33]		0.59	2.05	0.13 [0.06~0.37]	
	2–3 times a week	2.29	4.06	1.25 [0.53~2.32]		0.51	2.24	0.14 [0.06~0.35]		0.55	1.40	0.19 [0.08~0.44]	
	4–6 times a week	2.45	5.73	1.34 [0.57~2.47]		0.80	4.14	0.18 [0.06~0.42]		0.63	1.62	0.20 [0.09~0.48]	
	Once a day	2.92	9.89	1.11 [0.48~2.47]		0.58	1.70	0.17 [0.06~0.40]		0.55	2.31	0.14 [0.06~0.33]	
	Twice a day	2.36	4.15	1.26 [0.49~2.31]		0.57	1.46	0.20 [0.06~0.45]		0.41	0.85	0.17 [0.08~0.39]	
	Three a day	2.69	5.33	1.27 [0.56~2.58]		0.70	5.45	0.16 [0.06~0.41]		0.48	1.32	0.14 [0.06~0.36]	
PET bottle drink	Rarely	2.06	5.65	0.95 [0.37~1.99]	<0.001	0.45	1.44	0.16 [0.06~0.36]	0.030	0.45	1.71	0.13 [0.05~0.32]	<0.001
	Once a month	2.53	9.14	1.15 [0.43~2.24]		1.04	9.44	0.19 [0.06~0.42]		0.37	1.05	0.15 [0.06~0.32]	
	2–3 times a month	2.19	3.63	1.24 [0.54~2.43]		1.20	8.97	0.18 [0.06~0.45]		0.89	5.37	0.19 [0.07~0.48]	
	Once a week	2.48	4.56	1.30 [0.53~2.38]		0.61	2.61	0.16 [0.06~0.39]		0.64	2.21	0.14 [0.06~0.37]	
	2–3 times a week	3.02	8.29	1.33 [0.53~2.62]		1.01	6.61	0.17 [0.06~0.45]		0.53	1.49	0.17 [0.07~0.46]	
	4–6 times a week	2.57	5.19	1.38 [0.58~2.62]		0.99	4.81	0.22 [0.05~0.47]		1.09	3.40	0.21 [0.09~0.60]	
	Once a day	1.98	2.86	1.16 [0.44~2.15]		0.83	3.87	0.19 [0.06~0.45]		0.63	2.75	0.14 [0.06~0.41]	
	Twice a day	2.26	4.10	1.34 [0.57~2.13]		1.11	4.63	0.25 [0.10~0.49]		0.56	1.01	0.26 [0.12~0.61]	
	Three a day	2.64	6.65	1.14 [0.46~2.28]		0.54	1.90	0.16 [0.06~0.39]		0.45	1.31	0.14 [0.06~0.35]	

**Table 5:** Urinary bisphenol concentrations by cooking utensil use (e.g., coated cookware, rice cookers, etc.).

Variable		Bisphenol A (Urine, µg/L)				Bisphenol F (Urine, µg/L)				Bisphenol S (Urine, µg/L)			
		M	SD	Median (25–75%)	*p*-Value	M	SD	Median (25–75%)	*p*-Value	M	SD	Median (25–75%)	*p*-Value
Refrigerator Storage Containers	Glass bowl	2.30	5.40	1.11 [0.44~2.25]	0.346	0.68	4.12	0.16 [0.06~0.38]	0.063	0.53	1.92	0.15 [0.06~0.37]	0.191
	Metal bowl	2.28	3.77	1.44 [0.68~2.30]		0.73	3.83	0.15 [0.05~0.34]		0.39	0.74	0.16 [0.07~0.41]	
	Plastic bowl	2.44	6.57	1.16 [0.48~2.29]		0.77	5.12	0.17 [0.06~0.43]		0.57	2.69	0.15 [0.06~0.39]	
	Zipper bag/Plastic bag	2.55	3.28	1.73 [0.64~2.74]		0.38	0.49	0.20 [0.12~0.46]		0.99	2.31	0.30 [0.08~0.83]	
	ceramic bowl	2.82	7.60	1.27 [0.32~2.27]		0.46	0.87	0.23 [0.06~0.49]		0.60	3.33	0.15 [0.06~0.28]	
	Other	2.43		2.43 [2.43~2.43]		0.31		0.31 [0.31~0.31]		0.03		0.03 [0.03~0.03]	
Freezer Storage Containers	Glass bowl	1.34	1.09	1.16 [0.39~2.00]	0.612	0.87	1.89	0.27 [0.06~0.78]	0.612	0.64	0.92	0.33 [0.11~0.77]	<0.001
	Metal bowl	1.38	0.78	1.54 [0.88~1.66]		0.18	0.19	0.15 [0.03~0.21]		1.25	1.80	0.69 [0.16~0.96]	
	Plastic bowl	2.23	3.78	1.21 [0.55~2.32]		0.63	3.03	0.16 [0.06~0.43]		0.54	2.05	0.16 [0.07~0.4]	
	Zipper bag/Plastic bag	2.42	6.25	1.14 [0.44~2.27]		0.72	4.70	0.17 [0.06~0.40]		0.55	2.38	0.15 [0.06~0.37]	
	ceramic bowl	1.86	2.28	0.80 [0.04~4.31]		0.22	0.19	0.22 [0.04~0.33]		0.66	0.52	0.43 [0.25~1.23]	
	Other	2.85	2.49	2.43 [0.60~5.53]		0.43	0.40	0.31 [0.10~0.88]		0.09	0.07	0.07 [0.03~0.17]	
Frequency of storing hot, cooked food in hard transparent plastic containers before washing dishes:	Rarely	2.30	5.36	1.11 [0.44~2.23]	0.064	0.72	4.79	0.16 [0.06~0.39]	0.009	0.54	2.52	0.14 [0.06~0.35]	<0.001
	Once a month	2.15	3.68	1.09 [0.46~2.09]		0.36	0.61	0.20 [0.06~0.43]		0.68	1.89	0.19 [0.08~0.51]	
	2–3 times a month	2.52	4.82	1.38 [0.49~2.57]		0.73	3.44	0.18 [0.06~0.40]		0.58	1.08	0.19 [0.08~0.55]	
	Once a week	2.67	6.87	1.22 [0.57~2.41]		0.32	0.39	0.20 [0.06~0.42]		0.63	1.60	0.18 [0.08~0.48]	
	2–3 times a week	3.81	12.99	1.42 [0.60~2.88]		1.07	5.00	0.20 [0.06~0.47]		0.61	1.52	0.21 [0.08~0.5]	
	4–6 times a week	1.45	1.40	0.99 [0.48~1.95]		0.59	1.34	0.30 [0.10~0.58]		0.35	0.63	0.17 [0.08~0.31]	
	Once a day	1.95	2.88	1.15 [0.32~2.07]		1.01	4.38	0.23 [0.06~0.54]		0.58	1.43	0.16 [0.05~0.46]	
	Twice a day	1.79	0.70	2.01 [1.31~2.27]		1.06	1.12	0.66 [0.36~1.76]		0.33	0.09	0.37 [0.27~0.39]	
	Three a day	3.94	2.24	3.94 [2.36~5.53]		0.25	0.20	0.25 [0.10~0.39]		0.18	0.01	0.18 [0.17~0.18]	
Frequency of pouring boiling water into hard transparent plastic containers:	Rarely	2.39	6.11	1.14 [0.45~2.27]	0.434	0.71	4.58	0.16 [0.06~0.40]	0.006	0.55	2.37	0.15 [0.06~0.37]	<0.001
	Once a month	1.91	3.02	1.22 [0.50~1.93]		0.52	1.04	0.24 [0.10~0.51]		0.81	2.42	0.25 [0.13~0.69]	
	2–3 times a month	1.97	1.68	1.55 [0.77~2.60]		0.64	1.37	0.28 [0.11~0.51]		0.64	1.78	0.29 [0.1~0.56]	
	Once a week	2.16	2.89	1.18 [0.44~1.99]		0.29	0.29	0.25 [0.07~0.43]		0.51	0.76	0.19 [0.1~0.67]	
	2–3 times a week	2.55	4.52	1.05 [0.44~2.28]		1.68	6.62	0.33 [0.15~0.72]		0.57	1.21	0.21 [0.08~0.44]	
	4–6 times a week	1.47	0.84	1.47 [0.88~2.07]		0.50	0.67	0.50 [0.03~0.97]		0.04	0.03	0.04 [0.02~0.06]	
	Once a day	2.35	3.38	1.23 [0.62~2.35]		0.75	2.14	0.28 [0.12~0.47]		0.24	0.34	0.13 [0.08~0.18]	
	Twice a day	4.43	4.81	2.72 [1.53~7.33]		1.14	1.33	0.91 [0.03~2.25]		0.33	0.18	0.34 [0.21~0.44]	
	Three a day	5.53		5.53 [5.53~5.53]		0.10		0.10 [0.10~0.10]		0.17		0.17 [0.17~0.17]	
Use a coated frying pan	Rarely	3.11	6.11	0.90 [0.44~2.70]	0.008	1.06	3.29	0.22 [0.06~0.63]	0.164	0.50	1.05	0.14 [0.06~0.51]	0.001
	Once a month	2.45	3.91	1.13 [0.39~2.58]		0.77	1.84	0.23 [0.06~0.52]		0.38	0.50	0.19 [0.07~0.54]	
	2–3 times a month	3.12	5.99	1.40 [0.69~2.61]		0.47	1.23	0.21 [0.06~0.43]		0.95	3.47	0.18 [0.07~0.47]	
	Once a week	2.07	3.75	0.93 [0.39~2.08]		0.62	2.44	0.18 [0.06~0.42]		0.51	2.96	0.11 [0.06~0.29]	
	2–3 times a week	2.49	6.96	1.09 [0.44~2.30]		0.55	2.43	0.16 [0.06~0.39]		0.44	1.46	0.14 [0.06~0.34]	
	4–6 times a week	2.09	5.47	1.22 [0.51~2.20]		0.71	4.12	0.18 [0.06~0.42]		0.52	1.29	0.16 [0.07~0.44]	
	Once a day	2.32	5.96	1.11 [0.41~2.16]		0.96	7.07	0.15 [0.06~0.39]		0.56	2.96	0.14 [0.06~0.36]	
	Twice a day	2.39	4.77	1.29 [0.58~2.26]		0.53	1.38	0.18 [0.06~0.41]		0.96	3.41	0.19 [0.08~0.47]	
	Three a day	2.79	3.50	1.45 [0.87~3.85]		0.36	0.60	0.14 [0.03~0.33]		0.47	0.91	0.21 [0.06~0.47]	
Use a coated pot	Rarely	2.23	5.70	1.08 [0.44~2.18]	<0.001	0.78	5.59	0.17 [0.06~0.40]	0.529	0.41	1.32	0.13 [0.06~0.33]	<0.001
	Once a month	2.46	2.81	1.61 [0.79~2.82]		0.57	1.58	0.18 [0.05~0.42]		0.47	0.71	0.20 [0.09~0.41]	
	2–3 times a month	3.28	5.40	1.32 [0.69~3.17]		0.54	2.18	0.17 [0.06~0.37]		0.87	3.46	0.18 [0.07~0.49]	
	Once a week	2.03	2.51	1.36 [0.62~2.34]		0.71	3.35	0.16 [0.06~0.42]		0.74	2.29	0.17 [0.06~0.41]	
	2–3 times a week	2.54	5.69	1.07 [0.42~2.35]		0.45	1.44	0.18 [0.06~0.39]		0.58	2.33	0.15 [0.06~0.38]	
	4–6 times a week	2.23	6.66	1.22 [0.43~2.30]		1.21	6.27	0.20 [0.06~0.47]		0.64	1.66	0.19 [0.08~0.56]	
	Once a day	2.50	7.53	1.07 [0.41~2.04]		0.72	4.58	0.15 [0.06~0.40]		0.50	3.11	0.14 [0.05~0.33]	
	Twice a day	2.49	3.48	1.39 [0.71~2.66]		0.39	0.70	0.22 [0.06~0.42]		1.19	4.38	0.22 [0.11~0.53]	
	Three a day	2.48	3.33	1.45 [0.84~2.87]		0.36	0.66	0.07 [0.03~0.30]		0.47	0.84	0.31 [0.11~0.51]	
Use a coated container	Rarely	2.41	6.10	1.11 [0.45~2.27]	0.396	0.71	4.97	0.16 [0.06~0.39]	0.868	0.50	2.33	0.13 [0.06~0.33]	<0.001
	Once a month	2.15	3.70	1.17 [0.46~2.35]		0.44	0.92	0.18 [0.05~0.47]		0.59	1.36	0.17 [0.08~0.5]	
	2–3 times a month	2.12	3.38	1.19 [0.52~2.30]		0.70	2.97	0.22 [0.06~0.42]		0.83	2.67	0.21 [0.09~0.52]	
	Once a week	2.10	3.48	1.15 [0.41~2.13]		0.99	4.29	0.15 [0.06~0.37]		0.67	3.60	0.19 [0.07~0.40]	
	2–3 times a week	2.75	8.70	1.23 [0.52~2.20]		0.60	1.81	0.17 [0.06~0.44]		0.43	0.72	0.17 [0.07~0.47]	
	4–6 times a week	2.23	6.46	1.27 [0.53~2.23]		1.49	6.59	0.20 [0.05~0.52]		0.85	2.17	0.25 [0.11~0.63]	
	Once a day	2.40	5.67	0.95 [0.34~2.17]		0.44	1.06	0.17 [0.06~0.45]		0.74	1.96	0.19 [0.09~0.60]	
	Twice a day	2.32	2.82	1.71 [0.71~2.42]		0.24	0.23	0.12 [0.04~0.45]		1.89	4.92	0.43 [0.18~1.32]	
	Three a day	2.45	1.69	2.02 [1.25~3.85]		0.41	0.55	0.20 [0.03~0.64]		0.23	0.27	0.10 [0.04~0.46]	
Use a coated electric rice cooker, pressure cooker	Rarely	2.42	5.07	1.12 [0.48~2.18]	0.014	0.68	2.64	0.16 [0.06~0.37]	0.802	0.44	1.00	0.13 [0.06~0.36]	<0.001
	Once a month	2.36	4.04	1.24 [0.32~2.29]		0.37	0.68	0.20 [0.06~0.34]		0.47	0.70	0.16 [0.04~0.68]	
	2–3 times a month	3.85	6.23	1.72 [0.90~4.31]		0.58	1.80	0.20 [0.08~0.44]		0.96	1.63	0.30 [0.13~0.95]	
	Once a week	3.18	10.19	1.34 [0.49~2.58]		0.74	2.65	0.19 [0.06~0.43]		0.83	3.37	0.18 [0.05~0.53]	
	2–3 times a week	2.15	3.52	1.10 [0.45~2.12]		0.78	5.48	0.17 [0.06~0.44]		0.60	2.71	0.15 [0.06~0.36]	
	4–6 times a week	2.47	8.41	1.24 [0.52~2.30]		1.07	7.03	0.18 [0.06~0.42]		0.50	1.14	0.19 [0.08~0.49]	
	Once a day	2.37	5.79	1.08 [0.42~2.28]		0.63	3.96	0.16 [0.06~0.39]		0.51	2.63	0.13 [0.06~0.33]	
	Twice a day	1.84	3.91	1.23 [0.52~2.08]		0.35	0.73	0.18 [0.06~0.40]		0.51	1.29	0.19 [0.07~0.40]	
	Three a day	2.74	4.19	1.55 [0.66~2.62]		0.54	1.13	0.20 [0.03~0.56]		1.46	4.28	0.24 [0.08~0.64]	

**Table 6:** Spearman correlation coefficients between urinary bisphenol concentrations and biochemical and anthropometric indicators.

	BPF	BPS	HDL	TC	TG	ALT	AST	GGT	Serum Cr	Height	Weight	Waist	Systole	Diastole
BPA	0.009	0.037 *	0.014	−0.005	0.001	0.001	−0.015	0.013	0.044 *	0.037 *	0.033 *	−0.006	−0.048 **	−0.023
BPF	1	0.019	0.006	0.002	−0.005	−0.024	−0.042 *	−0.020	−0.028	0.014	0.005	−0.002	−0.011	0.000
BPS			1	−0.017	−0.015	−0.002	−0.010	−0.020	−0.011	−0.011	0.045 **	0.022	−0.005	0.006

* *p* < 0.05, ** *p* < 0.01.

**Table 7:** Weighted linear regression analysis of hepatic biomarkers on log-transformed bisphenol concentrations.

Independent Variable	AST (β [SE], *p*)	ALT (β [SE], *p*)	Creatinine (β [SE], *p*)
log BPA	0.123 (0.041), 0.003	0.176 (0.054), 0.001	0.018 (0.012), 0.142
log BPF	0.071 (0.039), 0.065	0.094 (0.050), 0.072	0.011 (0.011), 0.305
log BPS	0.097 (0.034), 0.004	0.124 (0.046), 0.007	0.020 (0.009), 0.030

BPA was significantly associated with both AST and ALT (*p* < 0.01). BPS showed positive associations, while BPF was marginally significant. *p*-value; weighted complex sample model.

**Table 8:** Weighted multiple linear regression including log-BPA, log-BPF, log-BPS simultaneously.

Independent Variable	AST (β [SE], *p*)	ALT (β [SE], *p*)	Creatinine (β [SE], *p*)
log BPA	0.102 (0.037), 0.006	0.157 (0.049), 0.002	0.015 (0.010), 0.128
log BPF	0.044 (0.036), 0.214	0.061 (0.045), 0.185	0.009 (0.009), 0.312
log BPS	0.053 (0.031), 0.089	0.072 (0.039), 0.067	0.014 (0.008), 0.076

Only BPA retained significance for AST and ALT after mutual adjustment.

## Data Availability

Restrictions apply to the availability of these data. Data were obtained from the National Institute of Environmental Research (NIER) and are available from the authors with the permission of NIER. National Institute of Environmental Research (NIER). Korean National Environmental Health Survey (K-Nehes IV, 2018–2020) Data User Guide. Incheon.

## Supplementary Materials

The following supporting information can be downloaded at: https://www.mdpi.com/article/10.3390/toxics13121027/s1, Table S1: General characteristics of study participants (N = 4239).

## Abbreviations

The following abbreviations are used in this manuscript:

ALT	Alanine aminotransferase
AST	Aspartate aminotransferase
BPA	Bisphenol A
BPF	Bisphenol F
BPS	Bisphenol S
DBP	Diastolic blood pressure
EFSA	European Food Safety Authority
FDA	Food and Drug Administration
GGT	Gamma-glutamyl transferase
HDL	High-density lipoprotein
SBP	Systolic blood pressure
Scr	Serum creatinine
TC	Total cholesterol
TG	Triglycerides
WHO	World Health Organization.

## References

[1] Eckert E.M., Di Cesare A., Kettner M.T., Arias-Andres M., Fontaneto D., Grossart H.P., Corno G. (2018). Microplastics Increase Impact of Treated Wastewater on Freshwater Microbial Community. Environ. Pollut..

[2] Cox K.D., Covernton G.A., Davies H.L., Dower J.F., Juanes F., Dudas S.E. (2019). Human Consumption of Microplastics. Environ. Sci. Technol..

[3] Karami A., Golieskardi A., Choo C.K., Larat V., Karbalaei S., Salamatinia B. (2018). Microplastic and Mesoplastic Contamination in Canned Sardines and Sprats. Sci. Total Environ..

[4] Toussaint B., Raffael B., Angers-Loustau A., Gilliland D., Kestens V., Petrillo M., Rio-Echevarria I.M., Van den Eede G. (2019). Review of micro-and nanoplastic contamination in the food chain. Food Addit. Contam. Part A.

[5] Song U., Park H. (2024). Plastic recycling in South Korea: Problems, challenges, and policy recommendations in the endemic era. J. Ecol. Environ..

[6] Ebere E.C., Wirnkor V.A., Ngozi V.E. (2019). Uptake of Microplastics by Plants: A Reason to Worry or to Be Happy?. World Sci. News.

[7] Geyer R., Jambeck J.R., Law K.L. (2017). Production, Use, and Fate of All Plastics Ever Made. Sci. Adv..

[8] Munyaneza J., Mboumba S., Gavamukulya Y., Muhirwa D., Nguyen T.V. (2022). A Review of Atmospheric Microplastics Pollution: In-Depth Sighting of Sources, Analytical Methods, Physiognomies, Transport and Risks. Sci. Total Environ..

[9] Chen G., Feng Q., Wang J. (2020). Mini-Review of Microplastics in the Atmosphere and Their Risks to Humans. Sci. Total Environ..

[10] Habibi N., Uddin S., Fowler S.W., Behbehani M. (2022). Microplastics in the Atmosphere: A Review. J. Environ. Expo. Assess..

[11] Lee H.S., Kim Y.J. (2017). Estimation of Microplastics Emission Potential in South Korea—For Primary Source. Sea J. Korean Soc. Oceanogr..

[12] Miyamoto K., Kotake M. (2006). Estimation of daily bisphenol a intake of Japanese individuals with emphasis on uncertainty and variability. Environ. Sci..

[13] World Health Organization (2019). Microplastics in Drinking-Water.

[14] Centre for Food Safety Food Safety Focus, Issue 143 (June 2018). https://www.cfs.gov.hk/english/multimedia/multimedia_pub/multimedia_pub_fsf.html.

[15] Liao C., Liu F., Alomirah H., Loi V.D., Mohd M.A., Moon H.B., Nakata H., Kannan K. (2012). Bisphenol S in urine from the United States and seven Asian countries: Occurrence and human exposures. Environ. Sci. Technol..

[16] Rochester J.R., Bolden A.L. (2015). Bisphenol S and F: A Systematic Review and Comparison of the Hormonal Activity of Bisphenol A Substitutes. Environ. Health Perspect..

[17] Rochester J.R. (2013). Bisphenol A and human health: A review of the literature. Reprod. Toxicol..

[18] Vandenberg L.N., Hauser R., Marcus M., Olea N., Welshons W.V. (2007). Human exposure to bisphenol A (BPA). Reprod. Toxicol..

[19] Lehmler H.J., Liu B., Gadogbe M., Bao W. (2018). Exposure to Bisphenol A, Bisphenol F, and Bisphenol S in U.S. Adults and Children: The National Health and Nutrition Examination Survey 2013–2014. ACS Omega.

[20] Lambré C., Barat Baviera J.M., Bolognesi C., Chesson A., Cocconcelli P.S., Crebelli R., Gott D.M., Grob K., Lampi E., EFSA Panel on Food Contact Materials, Enzymes and Processing Aids (CEP) (2023). Re-evaluation of the risks to public health related to the presence of bisphenol A (BPA) in foodstuffs. EFSA J..

[21] Rahman A., Sarkar A., Yadav O.P., Achari G., Slobodnik J. (2021). Potential Human Health Risks Due to Environmental Exposure to Nano- and Microplastics and Knowledge Gaps: A Scoping Review. Sci. Total Environ..

[22] Song Y.M., Kim C.Y. (2021). Toxicities Demonstrated in Dams and Neonates Following Intragastric Intubation of Polyethylene Microplastics to Pregnant Mice. J. Environ. Health Sci..

[23] Han Y., Song Y., Kim G.W., Ha C., Lee J., Kim M., Son H., Lee G., Gautam R., Heo Y. (2021). No Prominent Toxicity of Polyethylene Microplastics Observed in Neonatal Mice Following Intratracheal Instillation to Dams During Gestational and Neonatal Period. Toxicol. Res..

[24] Lv Y., Lu S., Dai Y., Rui C., Wang Y., Zhou Y., Li Y., Pang Q., Fan R. (2017). Higher dermal exposure of cashiers to BPA and its association with DNA oxidative damage. Environ. Int..

[25] Reale E., Vernez D., Hopf N.B. (2021). Skin Absorption of Bisphenol A and Its Alternatives in Thermal Paper. Ann. Work Expo. Health.

[26] Liao C., Kannan K. (2014). A Survey of Bisphenol A and Other Bisphenol Analogues in Foodstuffs from Nine Cities in China. Food Addit. Contam. Part A.

[27] Rudel R.A., Gray J.M., Engel C.L., Rawsthorne T.W., Dodson R.E., Ackerman J.M., Rizzo J., Nudelman J.L., Brody J.G. (2011). Food Packaging and Bisphenol A and Bis(2-ethylhexyl) Phthalate Exposure: Findings from a Dietary Intervention. Environ. Health Perspect..

[28] Liao C., Kannan K. (2013). Concentrations and profiles of bisphenol A and other bisphenol analogues in foodstuffs from the United States and their implications for human exposure. J. Agric. Food Chem..

[29] Geens T., Aerts D., Berthot C., Bourguignon J.P., Goeyens L., Lecomte P., Maghuin-Rogister G., Pironnet A.M., Pussemier L., Scippo M.L. (2012). A review of dietary and non-dietary exposure to bisphenol-A. Food Chem. Toxicol..

[30] Krivohlavek A., Benic N., Mihaljevic B., Dvorscak M. (2023). Migration of BPA from Food Packaging and Household Products on the Croatian Market. Int. J. Environ. Res. Public Health.

[31] Manzoor M.F., Ahmad N., Imran M., Rizwan M. (2022). An Insight into Bisphenol A, Food Exposure and Its Adverse Effects on Health: A Review. Front. Nutr..

[32] Gálvez-Ontiveros Y., Monteagudo C., Giles-Mancilla M., Muros J.J., Almazán V., Martínez-Burgos M.A., Samaniego-Sánchez C., Salcedo-Bellido I., Rivas A., Zafra-Gómez A. (2024). Dietary Bisphenols Exposure as an Influencing Factor of Body Mass Index. Environ. Health.

[33] Reininger N., Oehlmann J. (2024). Regrettable Substitution? Comparative Study of the Effect Profile of Bisphenol A and Eleven Analogues in an In Vitro Test Battery. Environ. Sci. Eur..

[34] Nelson J.W., Scammell M.K., Hatch E.E., Webster T.F. (2012). Social Disparities in Exposures to Bisphenol A and Polyfluoroalkyl Chemicals: A Cross-Sectional Study within NHANES 2003–2006. Environ. Health.

[35] Adamovsky O., Hilscherova K., Blaha L. (2024). Exploring BPA Alternatives–Environmental Levels and Toxicity Review. Environ. Int..

[36] National Toxicology Program (2008). NTP-CERHR Monograph on the Potential Human Reproductive and Developmental Effects of Bisphenol A.

[37] European Food Safety Authority (2015). Scientific Opinion on the Risks to Public Health Related to the Presence of Bisphenol A (BPA) in Foodstuffs. EFSA J..

[38] Lin Y.-J., Chen H.-C., Chang J.-W., Huang H.-B., Chang W.-T., Huang P.-C. (2024). Exposure Characteristics and Cumulative Risk Assessment of Bisphenol A and Its Substitutes: The Taiwan Environmental Survey for Toxicants 2013. Front. Public Health.

