# Supplementary material for: Associations of Lifestyle and Dietary Factors with Urinary Bisphenol A, S, and F: Evidence from the Korean National Environmental Health Survey IV (2018–2020)
Source: Toxics. 2025 Nov 27;13(12):1027. doi: 10.3390/toxics13121027 (PMC12737383; doi:10.3390/toxics13121027)
Supplement: Supplementary file 1 [file toxics-13-01027-s001.zip › toxics-3946148-supplementary.pdf]

Table S1. General characteristics of study participants (*N* = 4239).

Variable		Mean	SD	Median (25%-75%)	Range (μg/L)
Gender	Male (n, %)	1,889	44.6	-	-
	Female (n, %)	2,350	55.4	-	-
Height (cm)		162.64	8.91	162.00 [156.00-169.40]	133.30-191.20
Weight (kg)		65.90	12.62	64.30 [56.70-73.50]	40.00-136.30
Waist circumference (cm)		85.24	10.41	85.00 [78.00-92.00]	56.00-133.00
Systolic blood pressure (mmHg)		132.70	17.62	131 [120−144]	80.00-213.00
Diastolic blood pressure (mmHg)		79.55	12.43	79 [71−87]	40.00-140.00
Bisphenol A (urine, μg/L)		2.38	5.99	1.15 [0.45-2.27]	0.02-128.04
Bisphenol F (urine, μg/L)		0.71	4.52	0.17 [0.06-0.41]	0.03-142.48
Bisphenol S (urine, μg/L)		0.55	2.33	0.15 [0.06-0.38]	0.02-88.91
High-density lipoprotein cholesterol (mg/dL)		54.75	14.23	53 [44-63]	21.00-145.00
Total cholesterol (mg/dL)		188.21	38.04	187 [163-212]	80.00-655.00
Triglycerides (mg/dL)		182.58	149.03	144 [97-219]	29.00-3099.00
Alanine aminotransferase (ALT, U/L)		25.34	16.44	21 [16-29]	6.00-250.00
Aspartate aminotransferase (AST, U/L)		26.12	9.62	24 [21-29]	12.00-190.00
Gamma-glutamyl transferase (GGT, U/L)		31.32	39.78	20.00 [13.00-34.00]	2.40-623.00
Serum creatinine (mg/dL)		0.79	0.20	0.77 [0.66-0.90]	0.35-5.54
Average duration of exercise per session (min)		78.86	48.05	60 [60-90]	10.00-420.00
